# Effectiveness of Incentive Spirometry Versus Deep Breathing Exercises in Preventing Postoperative Pulmonary Complications After Abdominal Surgery: A Comprehensive Review

**DOI:** 10.7759/cureus.80149

**Published:** 2025-03-06

**Authors:** Qais M Ababneh, Hanaa Abdelrahman, Mohamed E Abdelhameed

**Affiliations:** 1 Intensive Care-Oncology, Jordan University of Science and Technology, Irbid, JOR; 2 Critical Care, HMS Mirdif Hospital, Dubai, ARE; 3 Emergency Medicine, Al Qassimi Hospital, Khartoum, SDN

**Keywords:** abdominal surgery, deep breathing exercises, incentive spirometry, postoperative pulmonary complications, respiratory function

## Abstract

This article review was conducted to evaluate the evidence of the use of an incentive spirometer (IS) versus deep breathing exercise (DBE) for the prevention of postoperative pulmonary complications (PPCs) after abdominal surgeries (upper and lower abdominal surgeries). Searches were performed in the following databases: PubMed, Medline, Embase, Web of Science, and Scopus to select articles and randomized controlled trials in which IS and DBE were used in the pre- and/or postoperative period in order to prevent PPCs and/or recover lung function after abdominal surgeries. The review concludes that implementing DBE and IS in the perioperative care of patients undergoing abdominal surgery is effective in preventing PPCs and improving recovery of pulmonary function. These findings support the integration of these techniques into standard postoperative care protocols to enhance patient outcomes following surgery with further recommendations to enhance patient compliance with IS.

## Introduction and background

Abdominal surgery encompasses a variety of surgical procedures performed in the abdominal region to diagnose and potentially address a specific medical issue [1]. The techniques and procedures employed can vary based on the abdominal organ affected and the condition being investigated. Traditionally, these surgeries necessitate a large incision to open the abdomen, which is known as open abdominal surgery or laparotomy [1,2]. Upper abdominal surgery refers to any surgical procedure conducted through an incision located at or above the level of the umbilicus. The recovery process following such surgery is typically complex and necessitates the involvement of multiple healthcare practitioners to ensure success. However, recovery itself is often an ambiguous concept, with both healthcare professionals and patients struggling to define it clearly [2].

Postoperative pulmonary complications (PPCs) are common among patients who undergo abdominal surgery, with reported rates ranging from 17% to 88% for upper abdominal procedures [3]. These complications can include conditions such as atelectasis, pneumonia, and respiratory failure. PPCs frequently occur in patients following abdominal surgery due to several factors, and the most commonly identified factors comprised an American Society of Anesthesiologists (ASA) score greater than III, old age (>65 years), a history of upper respiratory tract infections, the use of general anesthesia, the presence of comorbidities, smoking, alcoholism, low socioeconomic status, oxygen desaturation below 94%, serum albumin levels under 3.5 g/dL, intraoperative bleeding, hemoglobin levels below 10 g/dL, intraoperative blood transfusions, prolonged surgical procedures, postoperative mechanical ventilation, extended hospital stays, cardiac surgery, a history of respiratory diseases, inadequate postoperative pain control, the use of neuromuscular blocking agents, and delayed ambulation [4].

Abdominal surgeries are primarily conducted under general anesthesia, which can hinder the function of respiratory muscles. This impairment in respiratory muscle function results in decreased vital capacity (VC) and functional residual capacity (FRC) [5]. Pain at the surgical site in patients who have undergone upper abdominal surgery often leads to shallow and monotonous breathing. This altered breathing pattern can reduce ventilation in the lung regions that are dependent, potentially resulting in atelectasis [6]. The effects of anesthesia on the respiratory system include a depression of respiratory function, which impacts the cough reflex. Additionally, reduced lung volume, along with incision pain and the supine position, affects the mucociliary escalator, leading to an accumulation of secretions [5,7]. Physiotherapy plays a crucial role in aiding recovery from surgery, primarily by preventing or addressing postoperative complications and facilitating physical rehabilitation to help patients return to their presurgery functional levels [7]. While its main focus is on physical recovery, physiotherapy can also influence other aspects of well-being. The rehabilitation process begins before surgery and continues through the acute and subacute phases of recovery, often extending into community-based care after hospital discharge. This ongoing support is essential for helping individuals resume their normal daily activities and regain overall functionality [8].

Chest physiotherapy encompasses various techniques such as deep breathing exercises (DBEs), splinted active coughing, the use of incentive spirometry (IS), inspiratory muscle training, and education on the importance of early mobilization. Engaging in practical training enhances respiratory function before surgery and offers significant advantages in promoting lung expansion after the procedure compared to not implementing any intervention [7].

IS is a key component of care for postoperative and hospitalized patients, extensively researched in inpatient settings [8-10]. This device encourages patients to engage in slow, deep breathing by providing visual feedback, which aids in the expansion and reopening of collapsed airways. The use of an IS is advantageous because it is cost-effective, is easy to operate, has no known adverse effects, and does not require supervision once the patient has been adequately trained. Additionally, reaching the visual targets set by the device motivates patients to exert effort, thereby enhancing their compliance with the exercise [11].

Physiotherapists utilize breathing exercises to aid in the removal of secretions, enhance thoracic mobility, promote relaxation, manage breathlessness, increase pulmonary ventilation, and improve chest wall mobilization [12-14]. Various types of breathing exercises include diaphragmatic breathing, pursed lip breathing, segmental breathing, low-frequency breathing, sustained maximal inspiration, and exercises linked with postural adjustments. Additionally, resisted breathing programs, such as inspiratory resistive muscle training and abdominal weight training, are highly effective for respiratory training and enhancing pulmonary function [13].

There is still controversy about the clinical benefits of IS and DBE in the prevention of PPCs and mortality in adults undergoing abdominal surgeries. The effectiveness of IS and DBE remains debated due to several unresolved aspects including the following: First, there is inconclusive evidence regarding the clinical benefits of IS in reducing PPCs across various surgeries, with studies showing no significant advantage of IS over DBE or other physiotherapy techniques. Second, patient compliance with IS is a significant challenge, as adherence levels are often low and difficult to measure accurately, further confounding outcomes. Lastly, the lack of standardized protocols for IS use and the absence of robust data on its superiority over alternative strategies like early mobilization or directed coughing add to the uncertainty. Therefore, the objective of this work is to conduct an article review to evaluate the evidence on the use of DBE and IS in the prevention of PPCs and the recovery of pulmonary function in patients undergoing abdominal surgery.

## Review

Methods

Inclusion Criteria

The current study included articles and randomized controlled trials (RCTs) that utilized both DBEs and IS in preoperative and/or postoperative care, specifically aimed at reducing the rate of PPCs in individuals undergoing elective abdominal surgical procedures. Excluded from the study were narrative reviews, retrospective studies, non-controlled studies, personal communications, case reports, and any research that focused on using IS for training inspiratory muscles.

Measured Outcomes

Studies that assessed the following clinical outcomes were deemed eligible for inclusion in this research: pneumonia, atelectasis, pulmonary function, oxygenation, and the duration of hospital stays, provided that there was a follow-up of at least two days postoperatively.

Search Strategies and Selection

The search strategy involved conducting searches across several databases, including PubMed, Medline, Embase, Web of Science, and Scopus, targeting studies published up to December 2024. The search utilized specific terms such as “incentive spirometry,” “breathing exercise,” “chest physical therapy,” “respiratory therapy,” and “abdominal surgery.” Additionally, a follow-up search was performed by reviewing the reference lists of the studies identified in the initial search to uncover any additional relevant studies. Only studies published in English, Portuguese, and Spanish were included in the selection process.

Results

From 25 selected studies, only seven were considered to be included in this review published between 1997 and 2024. During the abstract screening, 18 studies were excluded, including 11 reviews, four studies with non-abdominal surgeries, one guideline, one in the pediatric field, and one published in another language. From the eight studies included in the analysis of the effect of DBE and IS in patients undergoing abdominal surgery, six studies (85.7%) were experimental studies and one study (14.3%) was a comparative study. Moreover, four studies (57.1%) had a control group without intervention and three studies (42.9%) did not, as shown in Table 1.

**Table 1:** Characteristics and results of the studies evaluating the effect of IS and DBE in abdominal surgeries VIS: volume-oriented incentive spirometry; FVC: forced vital capacity; FEV1: forced expiratory volume in one second; PEFR: peak expiratory flow rate; SpO_2_: peripheral oxygen saturation; PPCs: postoperative pulmonary complications; IS: incentive spirometry; DBE: deep breathing exercises; FIS: flow-based incentive spirometry; PEF: peak expiratory flow

Author (year)	Year	Design	Surgery	Intervention	Measurement	Outcomes
Othman et al. [15]	2016	Pilot randomized controlled trial	Lower abdominal surgery	IS (n = 20), DBE (n = 20)	Vital capacity (VC)	IS demonstrated greater objective improvement in VC than DBE. Both tools were valuable in PPCs (p = 0.52)
Sorour et al. [16]	2019	Quasi-experimental	Upper abdominal surgery	IS (n = 15), DBE (n = 15), control (n = 30)	PPC	Application of either DBE or IS proved to be equally influential in diminishing the incidence of PPC (p = 0.157)
Sushmitha et al. [17]	2024	Randomized controlled trial	Upper abdominal surgery	DBE (n = 15), IS (n = 15)	PEFR and level of dyspnea	There is an increase in the peak expiratory flow rate and a decrease in dyspnea values for both (p = 0.170)
Alaparthi et al. [18]	2016	Randomized controlled trial	Laparoscopic surgery	DBE (n = 65), FIS (n = 65), VIS (n = 65), control (n = 65)	FVC, FEV1, PEFR, PPCs	Pulmonary function (forced vital capacity) and diaphragm excursion showed statistically significant differences between volume incentive spirometry and diaphragmatic breathing exercise groups (p < 0.05) as compared to the flow incentive spirometry group and the control group
Shirodkar et al. [5]	2022	Randomized controlled trial	Upper abdominal surgery	DBE (n = 20), IS (n = 20)	PEFR, chest expansion, SpO_2_, PPCs	Peak expiratory flow rates, chest expansion, and pulse oximetry values improved in IS and DBE
Zhao et al. [19]	2023	Randomized clinical trial	Open abdominal surgery	DBE (n = 29), VIS (n = 29), control (n = 29)	FVC, PEF, FEV1, PEFR, PPCs	The significantly elevated levels of PEF, FEV1, FVC, and FEV1/FVC ratio were observed on the 1st, 3rd, and 5th postoperative days in the VIS and DBE groups compared to the control group (p < 0.05)
Lim et al. [20]	1997	Comparative study	Upper and lower abdominal surgeries	DBE (n = 30), IS (n = 30), control (n = 30)	PPCs	The frequency of development of pulmonary complications (43.3% in the control group, 20% in the DBE group, 20% in the IS group) (p < 0.05)

Table 1 summarizes a series of studies comparing the effectiveness of IS, DBEs, and other interventions in improving pulmonary outcomes following abdominal surgeries. These studies employed various designs, including RCTs, quasi-experimental designs, and comparative studies, to evaluate parameters such as pulmonary function, PPCs, and related metrics.

Studies varied in design, including RCTs, quasi-experimental, and comparative approaches, with sample sizes ranging from 15 to 65 participants. Smaller pilot studies, such as Othman et al. [15], Sorour et al. [16], and Sushmitha et al. [17], highlighted the potential benefits of IS in specific contexts [15]. RCTs by Alaparthi et al. [18] and Zhao et al. [19] provided robust evidence for volume-oriented IS (VIS) and DBE in enhancing postoperative pulmonary metrics. Control groups consistently exhibited poorer outcomes, reinforcing the necessity of postoperative respiratory interventions.

Heterogeneity in surgical procedures (e.g., upper vs. lower abdominal and laparoscopic vs. open) and measurement protocols (e.g., peak expiratory flow (PEF) rate (PEFR), forced expiratory volume in one second (FEV1), and dyspnea scales) may explain the variability in outcomes. For example, Shirodkar et al. [5] and Sorour et al. [16] focused on upper abdominal surgery but reported non-significant differences between interventions, whereas Alaparthi et al. emphasized laparoscopic contexts with significant results [18]. Additionally, older studies like Lim et al. [20] and newer trials like Sushmitha et al. [17] spanned over two decades, reflecting evolving methodologies.

A consistent theme across multiple studies was the superiority of both IS and DBE over control groups in reducing PPCs and enhancing pulmonary function. For instance, Alaparthi et al. further corroborated these findings, noting that VIS and DBE significantly improved forced VC (FVC) and diaphragm excursion compared to flow-based IS (FIS) and controls (p < 0.05) [18]. Zhao et al. observed elevated pulmonary function metrics (PEF, FEV1, and FVC) on postoperative days one, three, and five in the VIS and DBE groups versus controls (p < 0.05) [19]. Similarly, Lim et al. reported a significant reduction in PPCs with IS (20%) and DBE (20%) compared to controls (43.3%, p < 0.05) [20].

Studies evaluating objective pulmonary function metrics, such as FVC, FEV1, and PEFR, revealed mixed results. Alaparthi et al. [18] found that VIS and DBE significantly improved FVC and diaphragm excursion compared to the FIS and control groups (p < 0.05) in laparoscopic surgery. Zhao et al. [19] corroborated this, reporting elevated FVC, FEV1, and PEF levels in the DBE and VIS groups versus controls (p < 0.05) after open abdominal surgery. Conversely, Othman et al. noted greater improvements in VC with IS compared to DBE in lower abdominal surgery, though both interventions showed comparable efficacy in reducing PPCs (p = 0.52) [15]. Sushmitha et al. found no significant differences in PEFR or dyspnea reduction between DBE and IS (p = 0.170), suggesting functional equivalence in upper abdominal surgery [17].

Discussion

The review analyzed multiple RCTs that compared the effectiveness of IS and DBEs in preventing PPCs following abdominal surgeries. The studies included in the review varied in their methodologies, patient populations, and specific outcomes measured but collectively provided insights into the impact of these interventions.

PPCs refer to a range of adverse respiratory changes that can occur after surgery, significantly impacting patient recovery. The definitions of PPCs are established by the European Society of Anesthesiology and the European Society of Intensive Care Medicine [21,22].

Numerous literary sources have reported a significant variation in the incidence of PPCs, with rates ranging from 17% to 88% [3]. Variations in definitions, preoperative assessments to evaluate associated risks, diagnostic criteria, and the diversity of populations across different countries have all significantly contributed to the development of PPCs [20]. A study conducted in Nigeria found that the incidence of PPCs was 52%. In contrast, research from Zimbabwe and Ethiopia reported lower rates, with 42.4% and 21.7% of patients, respectively, experiencing these complications [23-26].

Postoperative pneumonia (PPC) can vary in severity, ranging from self-limiting cases to those requiring hospital interventions such as antibiotics or physiotherapy. In more severe instances, it may necessitate readmission to critical care or reintubation or can even lead to death [27]. The condition is associated with a significantly higher 30-day mortality rate of 18% compared to just 2.5% for patients without PPC. Furthermore, even after adjusting for risk factors, patients who experience PPC have a 66% lower survival rate five years postsurgery. For those who do survive, research suggests that PPC negatively impacts both early and long-term health-related quality of life. Following major elective abdominal surgery, patients with PPC typically incur an additional six to nine days in the hospital and impose an extra cost of approximately $30,000 on the healthcare system per patient [27].

In this review, IS and DBEs are both effective in reducing PPCs like atelectasis and pneumonia. Studies show that patients using these techniques experience fewer respiratory issues compared to those who do not [15,16]. While Othman et al. found that IS led to greater improvement in VC, they concluded that both IS and DBE are valuable tools in preventing PPCs [15]. Despite this study providing preliminary evidence favoring IS for VC improvement, its pilot nature and small sample size (n = 40) limit the generalizability of its findings.

Lim et al.’s study found that pulmonary complications were significantly more common in the control group (43.3%) compared to the DBE and IS groups (both 20%). However, the frequency of changes seen on chest X-rays was similar across all three groups: control (26.7%), DBE (16.7%), and IS (20%) [20].

Öner Cengiz et al. conducted a randomized controlled study with 66 participants to evaluate the impact of preoperative DBEs with an IS on respiratory parameters and complications in open-heart surgery patients. The participants were divided into a DBE group (n = 32) and a control group (n = 34). The deep breathing group performed DBEs with an IS before surgery, while the control group received standard hospital physiotherapy. The incidence of PPCs was significantly lower in the deep breathing group compared to the control group (3.1% vs. 23.5%, p < 0.05). Additionally, the deep breathing group exhibited significantly higher mean SpO_2_ values before surgery, on the first day in the Cardiovascular Surgery Unit, and at discharge compared to the control group (p-value < 0.05) [28].

IS and DBEs can lead to improved pulmonary function metrics, such as FVC and PEFR. A study by Shetty et al. indicated that pulmonary function and maximal respiratory pressures significantly increased across the board by the end of the intervention in all three groups: FIS, VIS, and DBE. However, the FIS and DBE groups showed better results than VIS, specifically in FVC (FIS group, 13.71%; VIS group, 14.89%; and DBE group, 21.27%), FEV1 (FIS group, 25.97%; VIS group, 22.52%; and DBE group, 19.38%), PEFR (FIS group, 38.76%; VIS group, 9.75%; and DBE group, 33.16%), maximum inspiratory pressure (MIP) (FIS group, 28.23%; VIS group, 19.36%; and DBE group, 52.14%), and maximum expiratory pressure (MEP) (FIS group, 43.00%; VIS group, 22.80%; and DBE group, 28.68%) [29].

Sushmitha et al. stated that there were statistically significant improvements in respiratory measurements like PEFR and levels of dyspnea after conducting both DBE and FIS [17]. In a study by Alaparthi et al., pulmonary function and diaphragm excursion significantly decreased on the first day after surgery across all groups, including IS, DBE, and a control group (p-value < 0.001). This decrease was more pronounced in the control group compared to the experimental groups. By the second postoperative day, VIS and DBEs better preserved pulmonary function (FVC) and diaphragm excursion compared to FIS and the control group. Statistically significant differences were found between the VIS and DBE groups compared to the FIS and control groups (p-value < 0.05) [18]. This study provides robust evidence due to its larger sample size (n = 260), but its focus on laparoscopic surgery may limit applicability to open or other types of abdominal surgeries.

Zhao et al. found that postoperatively, the IS and DBE groups exhibited reduced pulmonary function test values compared to their preoperative measurements; however, there was an improvement noted on the third and fifth days following surgery (p < 0.05). Notably, the VIS group showed significantly higher levels of PEF, FEV1, FVC, and the FEV1/FVC ratio on the first, third, and fifth postoperative days when compared to the control group (p < 0.05) [19]. This study's comprehensive measurement approach strengthens its conclusions but raises questions about whether VIS consistently outperforms other forms of spirometry or breathing exercises across different surgical contexts.

Clinical implications suggest that both DBE and IS are valuable, with selection guided by patient needs, surgical context, and resource availability. DBE, a low-cost, non-technical intervention, may enhance accessibility, while IS devices provide structured feedback, potentially aiding adherence. Future research should prioritize large-scale RCTs with standardized protocols, stratified by the surgery type and IS modality, to clarify optimal practices. Investigations into mechanistic differences-such as DBE’s impact on diaphragmatic strength versus IS’s role in alveolar recruitment-could further personalize postoperative care.

This comprehensive review of the current literature examines the use of IS and DBEs as effective measures for preventing PPCs following abdominal surgeries. The review synthesizes the existing current literature; however, the strength of our findings is inherently governed by the quality of the included manuscripts. Variability in study design, methodology, and reporting standards may influence the conclusions drawn.

The limitations of this article review stem from several factors that may affect the generalizability and robustness of its findings. First, the review included studies with a relatively small sample size, limiting the breadth of evidence available for analysis. Additionally, there was significant variability in the study designs, patient populations, and outcomes measured across the included studies, making it difficult to draw uniform conclusions. Another limitation is the focus on elective abdominal surgeries, which does not account for outcomes in emergency or more complex surgical cases. The studies also primarily assessed short-term outcomes, with limited exploration of long-term effects on pulmonary function or overall recovery.

## Conclusions

In summary, this review provides compelling evidence supporting the use of IS and DBEs as effective measures for preventing PPCs following abdominal surgeries. Both interventions contribute to improved pulmonary function and reduced hospital stays, highlighting their potential role in enhancing recovery processes.
